# Exploring long-term psychological effects of bronchiolitis and influenza in school-aged children

**DOI:** 10.3389/fped.2025.1536571

**Published:** 2025-03-10

**Authors:** Pankaj Soni, Jenny Cheriathu

**Affiliations:** ^1^ Department of Neonatology, Thumbay University Hospital, Ajman, United Arab Emirates; ^2^ Department of Clinical Sciences (Pediatric Neonatology), College of Medicine, Gulf Medical University, Ajman, United Arab Emirates; ^3^ Department of Pediatrics, Thumbay University Hospital, Ajman, United Arab Emirates

**Keywords:** bronchiolitis, influenza, mental health, psychological effects, school-aged children

## Abstract

**Introduction:**

This systematic review assessed the long-term psychological effects of severe respiratory infections—namely, bronchiolitis and influenza—in school-aged children (5–12 years).

**Methods:**

PubMed, EMBASE, and the Cochrane Library were searched for randomized controlled trials, cohort and longitudinal studies on school-aged children (5–12 years) with a history of bronchiolitis or influenza infection in early childhood published between 2014 and 2022. Studies evaluating long-term psychological outcomes at least 6 months post-infection were included.

**Results:**

Several studies that were included in this review reported increased risks of anxiety disorders, depression, and attention deficit among those with severe respiratory infections in early childhood. Additionally, studies with prolonged follow-up periods often reported a higher incidence of psychological morbidity in children. However, some studies did not detect significant long-term adverse effects, implying that timely interventions and supportive care may minimize negative psychological outcomes. This review underscores the necessity of mental health support following respiratory infections in children, highlights the need for further research on the biological and psychosocial pathways linking respiratory illnesses to psychological outcomes, and emphasizes the value of multidisciplinary treatment strategies for children with such comorbidities.

**Conclusions:**

The findings of this review provide insights for healthcare practitioners, policymakers, and researchers to consider strategies aimed at improving the outcomes of respiratory illnesses in affected children.

## 1 Introduction

Influenza and bronchiolitis are two of the most significant respiratory illnesses affecting children, with onset often occurring in infancy (1). Bronchiolitis, usually triggered by the respiratory syncytial virus, causes inflammation in the small airways of the lungs and may precipitate severe complications, including pneumonia (2, 3). Although short-term complications of respiratory illnesses are well documented, it is important to consider the long-term psychological impact on school-aged children who may have encountered respiratory illnesses during their infancy or toddler years (4, 5).

Recent epidemiological trends underscore the need for increased surveillance and scientific research. Notably, the characteristics of bronchiolitis infections may have changed post-COVID-19 pandemic, as highlighted in recent studies on bronchiolitis during the 2021–2023 epidemic seasons (6). Factors such as public health measures, competition among virus variants, and heightened awareness of respiratory illnesses have influenced both the severity and infection rates of bronchiolitis. Furthermore, new preventive strategies, such as nirsevimab (a monoclonal antibody targeting RSV), could alter future hospitalization rates and help prevent long-term sequelae (7).

The school-going age (5–12 years) is a developmental window characterized by rapid cognitive, emotional, and social growth. Children with chronic illnesses are at higher risk of psychological problems, such as anxiety, depression, and behavioral disorders (8). Although the physical consequences of bronchiolitis and influenza are well-studied, few studies examined their long-term psychological impact on children (9, 10). This knowledge gap is crucial, given that early-life respiratory diseases can influence developmental and psychosocial challenges later in life.

The current systematic review aims to summarize the evidence regarding the long-term psychological consequences of bronchiolitis and influenza in school-aged children. The core research questions include the following: (1) What psychological outcomes (e.g., anxiety, depression, behavioral, or cognitive problems) are associated with early-life bronchiolitis or influenza? (2) Are there specific socioeconomic or demographic factors (e.g., gender, household income, or maternal education) that modify these long-term psychological sequelae? (3) What preventive and clinical management policies can be implemented to provide holistic care interventions addressing both health and psychological issues in children with respiratory complaints?

### 1.1 Long-term psychological effects of bronchiolitis in a pediatric population

Several studies have examined post-bronchiolitis psychological outcomes in school-aged children. Martimbianco et al. (11) investigated the pulmonary and neurocognitive impact of severe bronchiolitis on children requiring mechanical ventilation. Their findings showed that survivors face intellectual and neurodevelopmental challenges later in life, including memory and attention deficits. Similarly, Freeman et al. (12) linked severe bronchiolitis to a higher risk of developing asthma and subsequent psychosocial stress. Contrarily, the findings of Wrotek et al. (13) showed no significant long-term psychological impact, such as depression and disruptive behavior, in most children hospitalized for bronchiolitis. This disparity between results could indicate variations in the severity of bronchiolitis, coping mechanisms, or socioeconomic and environmental factors across study populations.

### 1.2 Effects of influenza infection on developmental, cognitive, and behavioral outcomes

Influenza may also lead to various long-term psychological effects among children. Research conducted on adolescents with physical health issues, such as those who were discharged from medical facilities following influenza infection, demonstrated the occurrence of psychological distress and reduced cognitive performance (2). Endo et al. (14) studied classroom transmission of influenza in Japanese primary schools, observing disruptions in learning and social interactions due to school absenteeism. Moreover, a prospective observational study by Hoy et al. (15) demonstrated that annual influenza vaccination effectively minimized the severity of flu-related symptoms without showing any significant positive effects on long-term cognitive or psychological outcomes in school-aged children. Collectively, the findings of these studies suggest that although physical health might be acutely affected by influenza, residual psychological impacts can be subtle and may depend on vaccination coverage, timely treatment, and baseline health status.

### 1.3 Role of psychological support in respiratory illness outcomes

Literature underscores the importance of psychological interventions in children with respiratory conditions including bronchiolitis and influenza. Szakács et al. (9) described psychiatric comorbidities in children with post-H1N1 narcolepsy, emphasizing that early psychological support can forestall or mitigate further psychopathological developments. Similarly, Stevens and Kelsall-Knight (16) noted that children with asthma benefit from counseling, demonstrating reduced anxiety levels and improved quality of life. Not all studies, however, document robust integration of mental health services. Freeman et al. (12) highlighted that psychological symptoms are often neglected in children with severe respiratory infections, especially in developing countries. This underscores the importance of comprehensive care that acknowledges both physical and mental health components for pediatric patients.

## 2 Methods

### 2.1 Search strategy and data sources

A systematic review was conducted following the Preferred Reporting Items for Systematic Reviews and Meta-Analyses (PRISMA) guidelines. The literature search was conducted using PubMed, EMBASE, and the Cochrane Library. Reference lists of relevant articles were also examined. The review included randomized controlled trials (RCTs) and cohort and longitudinal studies on school-aged children (5–12 years) with a documented history of bronchiolitis or influenza infection in early childhood. Only studies evaluating long-term psychological outcomes—including anxiety, depression, cognitive function, or behavioral changes—at least 6 months post-infection were included. The search covered articles published between 2014 and 2022, without language restriction. Case reports, editorials, opinion pieces, or studies lacking explicit psychological or mental health outcomes, studies with follow-up periods of <6 months, and non-randomized trials were excluded. The keywords and search strings are presented in Table 1; study inclusion and exclusion criteria are listed in Table 2.

**Table 1:** Keywords and search strings.

Keywords/terms	Boolean operators	Search strings
Population		
“children,” “infants,” “pediatrics,” “preschool children”	OR	(children OR infants OR pediatrics OR preschool children)
Conditions		
“bronchiolitis,” “influenza,” “respiratory infection”	OR	(bronchiolitis OR influenza OR respiratory infection)
Outcomes		
“mental health,” “psychological,” “behavior”	OR	(mental health OR psychological OR behavior)
Combined search	AND	((children OR infants OR pediatrics OR preschool children) AND (bronchiolitis OR influenza OR respiratory infection) AND (mental health OR psychological OR behavior))

**Table 2:** Study eligibility criteria.

Criteria	Inclusion criteria	Exclusion criteria
Study design	Randomized controlled trials and cohort and longitudinal studies	Non-randomized studies, case reports, editorials, opinion pieces, and reviews
Population	School-aged children (5–12 years) with a history of bronchiolitis or influenza	Studies involving adults, adolescents (13+), or other populations not specific to school-aged children
Intervention/exposure	Children diagnosed with bronchiolitis or influenza infection	Studies focusing on other respiratory illnesses or unrelated diseases
Outcomes	Psychological outcomes (e.g., anxiety, depression, behavioral issues, cognitive outcomes)	Studies without specific psychological or mental health outcomes
Time frame	Studies that evaluate long-term psychological outcomes post-infection, with a minimum follow-up period of 6 months	Short-term studies with follow-up periods of <6 months
Language	No language restrictions, with translated articles being included when necessary	None
Publication date	Studies published from 2014 to 2022	Studies published before 2014
Full-text availability	Full-text articles available for review	Abstract-only publications or incomplete studies

### 2.2 Study selection

All references were imported into EndNote (Clarivate Analytics) for deduplication. Two independent reviewers (PS and JC) screened the titles and abstracts using Rayyan, a web-based screening tool. Full texts of potentially relevant studies were retrieved and reviewed thoroughly. Discrepancies were resolved through discussion until a consensus was reached. The final set of included studies was confirmed by both authors.

### 2.3 Data extraction

For data extraction, all identified abstracts and article citations were first reviewed for inclusion in this systematic review. A standardized MS Excel spreadsheet was created to capture relevant data from each included article, such as study characteristics (author, year of publication, design, and location), population details (participant age, sample size, and inclusion/exclusion criteria), exposure details (severity and timing of bronchiolitis or influenza infection), psychological outcomes (anxiety, depression, cognitive function, and behavioral impacts), follow-up duration, and key findings related to long-term mental health. All extracted data were checked for consistency and accuracy by both authors. This was supplemented with a careful analysis of the full text of each article to optimize the identification of all necessary data. PS performed the initial data extraction, which was independently validated by JC for accuracy. Any disagreements were reconciled through discussion. A PRISMA flowchart was developed to depict the selection process of studies included or excluded and the number of articles screened and included, along with the reasons for exclusions at each stage.

### 2.4 Risk of bias assessment

We employed the Cochrane risk of bias tool for RCTs and the Newcastle–Ottawa scale for observational studies, evaluating domains such as selection, performance, detection, and attrition bias. Studies were categorized as having a low, moderate, or high risk of bias.

## 3 Results

### 3.1 Study selection

**Figure 1:**
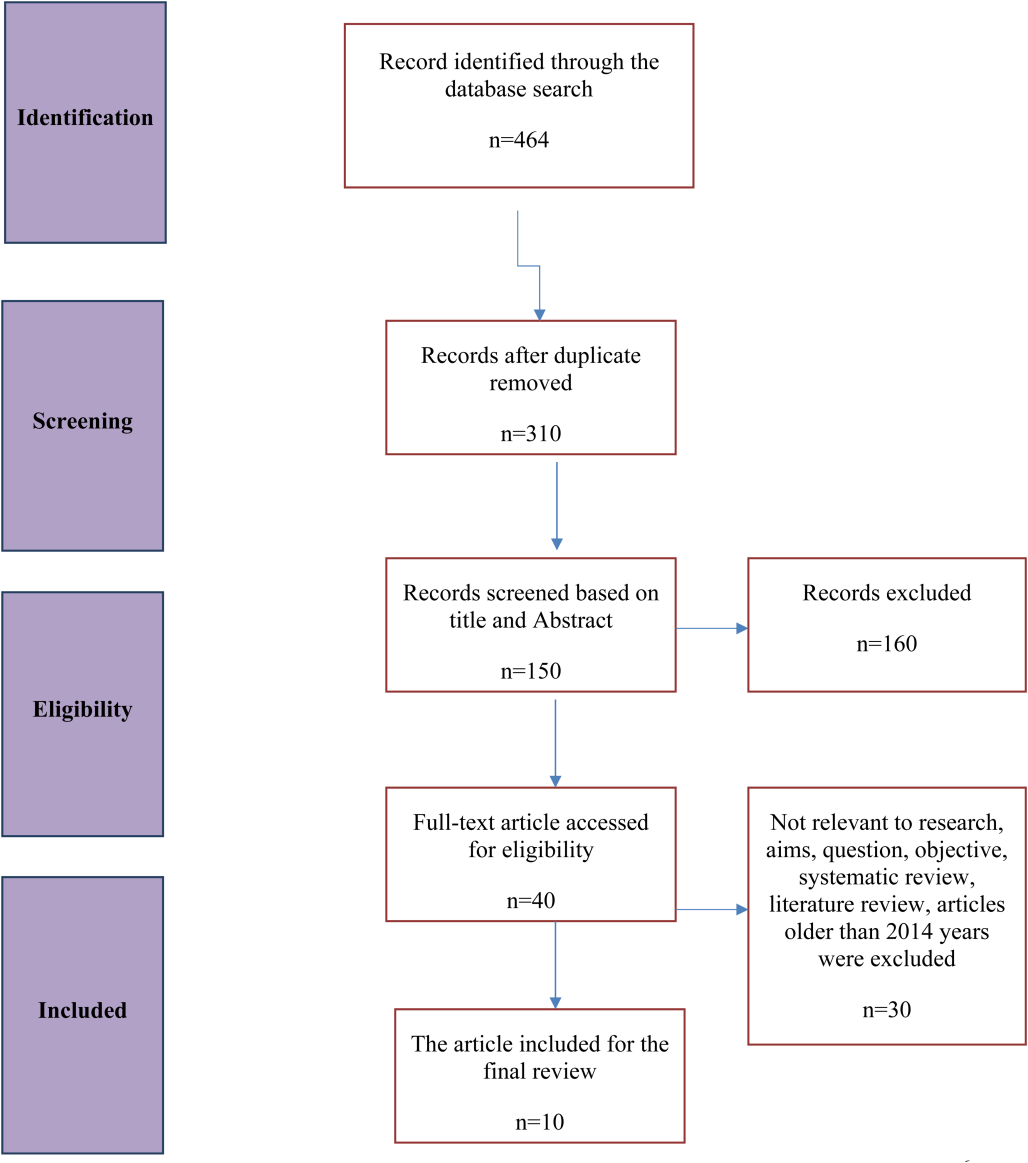
Flow chart of systematic literature search and article selection process.

A total of 464 articles were retrieved from the initial database search. After removing duplicates and articles that did not meet the initial screening criteria, 310 articles remained. Of these, 160 were excluded for being published outside the 2014–2022 timeframe or for lacking relevance to the study objectives. The full texts of the remaining 150 articles were reviewed against the inclusion and exclusion criteria, yielding 40 articles selected for detailed evaluation. Finally, 10 articles met all the eligibility criteria and were included in the qualitative synthesis (Figure 1).

### 3.2 Quality appraisal using the Cochrane Risk Of Bias Tool

The Cochrane risk of bias tool was used to assess the risk of bias and the quality of studies (11). The analysis revealed considerable variation in bias risk, necessitating careful interpretation of results. Most studies demonstrated low to moderate selection bias due to the use of matched cohorts and rigorous recruitment methods aimed at reducing confounding variables (14, 17). However, some studies relied on hospital-based samples, introducing a higher risk of selection bias and limiting the generalizability of findings (4). The risk of performance and detection biases was low for most studies, reflecting standardized treatment protocols and objective assessments (Table 3). However, some studies (18) were categorized as having higher risks due to non-blinded machine learning evaluations. Furthermore, attrition bias remained a concern in several studies where dropout rates and participant retention strategies were inadequately described (2, 19). Overall, although most studies provide valuable information, the presence of potential biases raises questions about the general and specific implications of the findings, emphasizing the need for further research to replicate conclusions in diverse samples.

**Table 3:** Cochrane risk of bias tool for quality appraisal.

Study	Selection bias	Performance bias	Detection bias	Attrition bias
Benjamin-Chung et al. (17)	Low - Matched cohort design can minimize bias	Low - Standard vaccination protocols likely used	Moderate - Laboratory confirmation may vary	Moderate - Not reported
de Sonnaville et al. (20)	Moderate - Selection of participants may influence outcomes	Low - Likely uniform treatment in the ICU	Low - Objective measurements (pulmonary outcomes)	Moderate - Dropout rates not detailed
de Sonnaville et al. (18)	Moderate - Potential bias if not all patients included	High - No blinding in machine learning assessment	Moderate - May vary across neurocognitive assessments	Moderate - Retention not discussed
Endo et al. (14)	Low - Population-based study reduces bias	Low - Consistent classroom environment	Low - Outcome assessment likely standardized	Moderate - Not specified
Ferro (2)	Low - Comparison group enhances selection validity	Moderate - Potential variability in psychological assessments	Moderate - Psychological measures may differ	High - Dropout rates not reported
McLean et al. (8)	Low - Population-based design	Low - Treatment protocols likely standardized	Moderate - Laboratory confirmation consistent	Moderate - Not mentioned
Sahoo and Ghosh (4)	High - Potential bias in the selection of hospitalized children	Moderate - Treatment protocols not specified	Moderate - Varying assessments may introduce bias	High - Attrition rates not reported
Szakács et al. (9)	Low - Controlled design minimizes bias	Moderate - Treatment protocols may differ	High - Varying assessments across cohorts	Moderate - Not detailed
Tunde-Ayinmode (19)	Moderate - Selection from a specific hospital	Moderate - Treatment may vary by protocol	Moderate - Assessments not standardized	High - Not mentioned
Uchida et al. (21)	Low - Prospective design reduces selection bias	Low - Participants likely treated uniformly	Moderate - Symptoms may vary based on reporting	Moderate - Dropout rates not specified

### 3.3 Anxiety

Anxiety is the main psychological outcome in children experiencing respiratory infections and those receiving care in the ICU (22). Sahoo and Ghosh (4) conducted a cross-sectional study on children with severe lower respiratory tract infections (LRTIs) in a tertiary care hospital in Eastern India. They found that 78.5% of children experienced delays in receiving medical attention. The delay was more pronounced in children of illiterate mothers compared to those of literate mothers. This delay acts as a stressor for both children and their caregivers due to the unpredictability of the child's condition or worsening of symptoms. Additionally, 67% of families belonged to the lowest socioeconomic strata, a factor that likely heightened anxiety due to financial constraints and inadequate access to healthcare (Table 4).

**Table 4:** Summary of main findings.

Author(s)	Method	Main findings	Sample size	Type of infection discussed
1. Sahoo and Ghosh (4)	Cross-sectional study in a tertiary care teaching hospital	Delays in seeking treatment may lead to prolonged illness and extended hospital stays, potentially causing long-term psychological stress, anxiety, and emotional trauma in children	42	Severe lower respiratory tract infection (LRTI)
2. de Sonnaville et al. (20)	Single-center cohort study	Children with a history of severe bronchiolitis and mechanical ventilation may experience psychological effects linked to chronic respiratory conditions like asthma. Frequent use of medication, ongoing medical interventions, and difficulty in physical activities could lead to anxiety, reduced self-esteem, and limitations in social participation	74	Severe bronchiolitis
3. de Sonnaville et al. (18)	Cross-sectional observational study	Lower intelligence, poorer attention and memory in children; factors like birth weight, socioeconomic status, and acidotic events impact neurocognitive outcomes	65	Bronchiolitis
4. de Sonnaville et al. (18)	Comparative study using neurocognitive testing	Children admitted to the pediatric intensive care unit for bronchiolitis requiring mechanical ventilation showed poorer neurocognitive outcomes (lower intelligence and attention and memory deficits). These deficits may lead to long-term psychological impacts like anxiety, learning difficulties, and lowered self-esteem	65	Bronchiolitis
5. Uchida et al. (21)	Prospective observational study	The study does not directly address long-term psychological impacts but notes that vaccinated children had reduced hospitalization rates, potentially mitigating anxiety and stress associated with severe illness and hospitalization	2,548	Influenza
6. Benjamin-Chung et al. (17)	Cohort study	Reducing illness-related absenteeism through school-based vaccination programs could positively impact children's mental health by reducing school disruption, promoting a sense of normalcy and alleviating anxieties associated with illnesses and absenteeism	13,217	Influenza
7. McLean et al. (8)	Prospective cohort study using multivariable logistic regression analysis	The study did not directly address psychological impacts. However, children with asthma who experience severe influenza may develop long-term psychological stress and anxiety related to chronic disease management and the fear of future severe episodes	1,764	Influenza
8. Szakács et al. (9)	Population-based, cross-sectional study using a variety of diagnostic tools (DSM-IV, ADHD rating scales, cognitive tests)	43% of patients with post-H1N1 narcolepsy had psychiatric comorbidities; cognitive assessments showed lower verbal comprehension and working memory	38 patients	H1N1 influenza (narcolepsy post-vaccination)
9. Tunde-Ayinmode (19)	Seventy-five (75) children aged 7–14 years with bronchial asthma were assessed using the Child Behavior Questionnaire and a semi-structured questionnaire	Probable psychological morbidity was present in 25% of the children. - Reported social impairments: 1. Interference with play: 60% 2. Interference with domestic work: 49% 3. Fear of dying: 29% 4. Feeling of being a burden: 25% - Significant associations with: 1. Lower maternal education (*p* = 0.020) 2. Maternal occupation (*p* = 0.038) 3. Polygamous family structure (*p* = 0.012) 4. Fathers having >5 children (*p* = 0.027) 5. Inadequate spousal support (*p* = 0.012)	75 children	Bronchial asthma
10. Ferro (2)	Data from the British Household Panel Survey (1991–2009) was used to compare healthy 16–25-year-olds (*n* = 7,342) with individuals with asthma (*n* = 1,798) and epilepsy (*n* = 117). Psychological distress was measured using the 12-item General Health Questionnaire	Prevalence ratios for psychological distress compared to healthy individuals ranged from 1.1 to 2.7 for asthma and 2.3 to 7.0 for epilepsy. - Risk for psychological distress among individuals with asthma (OR = 1.94, *p* < 0.0001) was significantly increased compared to healthy controls	7,342 healthy individuals, 1,798 with asthma, 117 with epilepsy	Asthma and epilepsy

Furthermore, children with a history of respiratory infections, particularly those requiring intensive care may experience long-term effects that further contribute to anxiety. In a single-center cohort study, de Sonnaville et al. (20) examined children aged 6–12 years who had undergone invasive mechanical ventilation for bronchiolitis. Of the 74 children treated, 26% had adverse long-term pulmonary outcomes, including asthma in 19% of patients (Table 4). A family history of atopic diseases increased the risk of asthma by approximately 6.4-fold, while prolonged mechanical ventilation was associated with worsened pulmonary outcomes. These long-term health concerns heighten parental stress, particularly regarding their child’s future health, physical activity limitations, and academic performance (23).

Another factor that explains the anxiety of affected families is the interaction between respiratory disorders and neurocognitive outcomes. de Sonnaville et al. (18) found that children with bronchiolitis requiring admission to the pediatric intensive care unit showed lower intelligence quotient (IQ) and poorer neurodevelopment than their healthy peers. These children also demonstrated deficits in attention and processing speed (MA = 54, *p* = 0.03). Additionally, lower birth weight and socioeconomic disadvantage were associated with reduced cognitive function, intensifying parental concerns about their child's educational and social prospects. Further research is necessary to explore the correlation between respiratory health, the emotional state of children and caregivers, and long-term psychological effects on children.

### 3.4 Depression

Bronchiolitis, as a respiratory infection, has been associated with psychological consequences, including depression and anxiety (24). A study conducted by Uchida et al. (21) examined the impact of influenza vaccination on symptom severity in children diagnosed with influenza and explored the broader mental health effects of respiratory diseases. This study included data from 13,217 children from elementary schools, of whom, 2,548 had influenza, 1,122 were vaccinated, and 1,426 were not (Table 4). Although both groups exhibited similar symptom frequency and fever duration, vaccinated children had significantly lower hospitalization rates, suggesting that vaccination may mitigate the severity of influenza-related symptoms. The study also calls for addressing both the physical and psychological health implications of respiratory infections.

Moreover, Benjamin-Chung et al. (17) discussed the effectiveness of a school-based influenza vaccination program in Oakland, California, assessing vaccination uptake, school absenteeism, and laboratory-confirmed influenza cases. Approximately, 7,502–10,006 (22%–28%) students were vaccinated annually. During the two subsequent influenza seasons 2016–2017 and 2017–2018, the intervention school group demonstrated increased vaccination coverage compared to the control group, which translated to a reduced hospitalization rate among vaccinated students. The intervention group demonstrated increased vaccination coverage compared to control schools, which translated to a reduced hospitalization rate among vaccinated students (Table 4).

Ferro (2) conducted a cross-sectional comparative study on psychological distress among adolescents and young adults with physical illnesses, such as asthma or epilepsy. This study compared data from 7,342 healthy adults with data from 1,798 with asthma and 117 with epilepsy. Both patient categories had a higher risk of psychological distress. People with asthma experienced 1.94 times higher odds of psychological distress than those without asthma (*p* < 0.0001) (Table 4).

These studies show that respiratory infections impact physical and mental health, with high rates of clinically diagnosed depression and anxiety observed in affected populations. Implementing preventive measures such as vaccination and early intervention could significantly mitigate the psychological impact associated with these respiratory diseases (25).

### 3.5 Psychiatric comorbidities including ADHD and cognitive effects

Medical interventions, including vaccines, may be associated with psychiatric conditions such as attention deficit hyperactivity disorder (ADHD) (26). A study conducted by Szakács et al. (9) enrolled patients with narcolepsy, which developed in a few people after H1N1 vaccination. Of the 38 patients evaluated, 43% in the vaccinated group had psychiatric comorbidities, including 29% with ADHD (inattentive type); 20% with mood disorders, particularly major depressive disorder; and 10% with general anxiety disorder. Furthermore, temper tantrums were noted at a high frequency (94%) in the vaccinated group (Table 4).

Although mean full-scale IQ scores remained within the normal range for both vaccinated and unvaccinated groups, individuals with psychiatric comorbidities demonstrated lower IQ scores than those without such conditions (9). This indicates that despite the absence of apparent cognitive decline in some patients, active psychiatric disorders can negatively impact cognitive functioning, including verbal comprehension and working memory. This impairment may be influenced by specific medical interventions, including vaccinations.

McLean et al. (8) compared the severity of confirmed laboratory cases of influenza infection among school-aged children with and without asthma. Among 1,764 children with medically attended influenza, 287 (16%) had confirmed asthma (Table 4). The study also concluded that the incidence rate of severe complications from influenza was the same for children with and without asthma. Children with asthma were 1.35 times more likely to be hospitalized or suffer from pneumonia than children without asthma (AOR 1.35). However, children with respiratory disorders should not be automatically assumed to be at greater risk of severe influenza and its complications. Additionally, their mental health should be monitored as they cope with chronic diseases.

Tunde-Ayinmode (19) conducted a study involving 75 children aged 7–14 years diagnosed with bronchial asthma attending clinics at the University of Ilorin Teaching Hospital in Nigeria. The assessment included the Child Behavior Questionnaire and a semi-structured questionnaire to determine psychological and social factors associated with asthma. The findings showed that 25% of children presented with probable psychological morbidity. The study also established that asthma had the following social impact: limitation in play (60% of respondents), disruption of domestic work (49%), fear of dying (29%), and feeling like a burden to the family (25%). Moreover, psychological morbidity was associated with maternal education (*p* = 0.020), maternal occupation (*p* = 0.038), polygamy (*p* = 0.012), and spousal support (*p* = 0.012). Logistic regression analysis revealed that perceived inadequate spousal support and low maternal occupational levels were additional predisposing factors for psychological morbidity in these children (Table 4).

## 4 Discussion

The analyzed studies indicate a potential connection between severe respiratory infections in early childhood and higher risks of mental health disturbances among school-aged children, such as anxiety, depression, and behavioral problems. Despite varied evidence, the literature consistently suggested a complex interplay involving pathophysiological and psychosocial factors. Systemic inflammation due to severe bronchiolitis or influenza infection may affect the developing central nervous system, leading to further consequences (8, 10). Factors like prolonged hospital stay, school absenteeism, and stress of parents may significantly influence a child's emotional and behavioral development (14).

According to Minotti et al. (27), despite being a pediatric health problem, bronchial asthma has a strong impact on psychosocial concerns, including perceiving life as a burden to the family and hindering daily activities. Similar to our findings, Ferro (2) found that adolescents with asthma have higher levels of psychological issues than their healthy peers. This highlights the need for both physical and mental evaluations of children with asthma, thereby guiding consensus on treatment and support interventions (28). The negative impact of low maternal education and occupational status on the psychological morbidity of children with asthma underscores the critical role of socioeconomic factors on health (29). Families with lower educational levels may be unaware of available health information and services that could help manage the psychological aspects of the disease (30). Hence, measures to ensure higher education for mothers and support structures could significantly reduce these psychological effects and improve the quality of life of children with asthma. Furthermore, self-reported social impairments emphasize the public sector consequences of providing care to children with chronic illnesses, thereby presenting comprehensive, family-focused community and policy solutions.

Moreover, the analysis reveals school absence resulting from respiratory ailments, highlighted in several studies (17, 21), necessitating preventive measures such as vaccination. Vaccination reduces hospitalization rates and ensures that children attend school and develop socially (31). Such impact of school absenteeism on quality of life and education further supports health promotion policies (32, 33).

Our findings have various implications for clinical practice and policy. For instance, integrating mental health screenings using standardized anxiety or depression scales during routine pediatric respiratory follow-ups may facilitate timely psychological evaluation (16). Other community-level interventions could also mitigate the negative impacts of illness on a child's academic and social well-being by strengthening family education and enhancing primary care resources. Furthermore, the standardization of outcome measures in longitudinal studies will provide clear insight into long-term effects and identify modifiable risk factors (9). All these coordinated efforts may lead to an integrated approach to addressing the problems prevalent among children with asthma and other chronic diseases, thus enhancing their quality of life and social inclusion.

## 5 Conclusion

This review reveals that bronchiolitis and influenza infection in early childhood can lead to long-lasting psychological impacts, including anxiety, depression, and attention deficits. However, timely interventions—such as vaccination—may offer significant protective benefits. Moreover, socioeconomic and demographic variables (e.g., maternal education and family structure) play a critical role. Additionally, the delayed or limited availability of psychological care—especially in resource-limited settings—underscores the importance of implementing interventions focused on the mental health of children. In conclusion, this literature review highlights that to optimize care for children recovering from respiratory illnesses, clinicians, policymakers, and educators must work together to develop comprehensive, multidisciplinary strategies that address the entire spectrum of health needs: physical, psychological, and social.

## Data Availability

The original contributions presented in the study are included in the article/Supplementary Material; further inquiries can be directed to the corresponding author.
